# Luminescent Conjugated Oligothiophenes for Sensitive Fluorescent Assignment of Protein Inclusion Bodies

**DOI:** 10.1002/cbic.201200731

**Published:** 2013-02-28

**Authors:** Therése Klingstedt, Cristiane Blechschmidt, Anna Nogalska, Stefan Prokop, Bo Häggqvist, Olof Danielsson, W King Engel, Valerie Askanas, Frank L Heppner, K Peter R Nilsson

**Affiliations:** [a] Department of Chemistry, Linköping University581 83 Linköping (Sweden) E-mail: petni@ifm.liu.se; [b] Department of Neuropathology, Charité-Universitätsmedizin BerlinCharitéplatz 1, 10117 Berlin (Germany); [c] Department of Neurology, USC Neuromuscular Center, University of Southern California Keck School of Medicine, Good Samaritan Hospital637 S. Lucas Ave, Los Angeles, CA 90017-1912 (USA); [d] Department of Neurology and Department of Clinical and Experimental Medicine, Division of Neurology, Faculty of Health Sciences, Linköping University581 83 Linköping (Sweden)

**Keywords:** amyloid beta-peptides, biosensors, fluorescent probes, luminescent conjugated oligothiophene, protein inclusion body

## Abstract

Small hydrophobic ligands identifying intracellular protein deposits are of great interest, as protein inclusion bodies are the pathological hallmark of several degenerative diseases. Here we report that fluorescent amyloid ligands, termed luminescent conjugated oligothiophenes (LCOs), rapidly and with high sensitivity detect protein inclusion bodies in skeletal muscle tissue from patients with sporadic inclusion body myositis (s-IBM). LCOs having a conjugated backbone of at least five thiophene units emitted strong fluorescence upon binding, and showed co-localization with proteins reported to accumulate in s-IBM protein inclusion bodies. Compared with conventional amyloid ligands, LCOs identified a larger fraction of immunopositive inclusion bodies. When the conjugated thiophene backbone was extended with terminal carboxyl groups, the LCO revealed striking spectral differences between distinct protein inclusion bodies. We conclude that 1) LCOs are sensitive, rapid and powerful tools for identifying protein inclusion bodies and 2) LCOs identify a wider range of protein inclusion bodies than conventional amyloid ligands.

## Introduction

Extracellular deposits of amyloidogenic, misfolded protein aggregates are the pathological hallmark of several degenerative diseases, and the development of amyloid-specific ligands to identify and characterize these protein deposits is therefore of great importance. Conventional amyloid ligands, such as derivatives of Congo Red and Thioflavin T, are rather selective for protein aggregates having an extensive cross β-pleated sheet conformation and sufficient structural regularity,^1^ but misfolded proteins do not always fulfill these requirements. Recently, luminescent conjugated oligo- and polythiophenes (LCOs and LCPs) were introduced as a novel class of amyloid-imaging ligands with the ability to identify and differentiate a broader subset of protein aggregates compared to conventional dyes.^2^–^12^ These thiophene-based ligands adapt their flexible conjugated backbones to the molecular structure of the deposits, and this adaptation is observed as a protein-structure-dependent change in the fluorescence emitted by the dye. Hence, heterogeneous populations of protein aggregates can be distinguished by spectral assignment. Chemically defined LCOs can also be utilized for real-time multiphoton in vivo imaging of protein aggregates, and show greater sensitivity and specificity than polydispersed LCPs towards protein aggregates.^8^

Protein aggregates can also be observed as intracellular inclusion bodies. Neurofibrillary tangles in Alzheimer’s disease^13^ (AD), Lewy bodies in Parkinson’s disease,^14^ and Pick bodies in Pick’s disease^15^ are all examples of neuronal inclusion bodies, but assembly of protein aggregates can also be seen in other cell types, such as hepatocytes^16^ and skeletal muscle fibers.^17^, ^18^ The latter is a pathological hallmark in sporadic inclusion body myositis (s-IBM), which is the most common myopathy in individuals over 50 years and is characterized by slowly progressing muscle weakness and atrophy.^18^ The inclusion bodies in s-IBM muscle fibers have been shown to consist of a wide variety of proteins, such as Aβ, phosphorylated tau, TAR DNA-binding protein 43 (TDP-43), α-synuclein, and prion protein.^19^–^23^ Recently, the multifunctional protein p62 was also reported to be an integral part of s-IBM inclusion bodies, and p62 immunoreactivity has been proposed as a specific diagnostic marker for s-IBM.^24^ Fluorescence-based detection of inclusion bodies binding Congo Red in skeletal muscle fibers is presently used as one of several methods in the clinical diagnosis of s-IBM.^25^ However, the information obtained from Congo Red and other conventional amyloid-binding agents is somewhat limited. Their structurally rigid backbone only offers an on-or-off mode, and the conformational heterogeneity reported for many amyloid proteins^26^ goes undetected. The LCO technique was recently used to gain novel molecular insights into hepatic protein inclusion bodies (Mallory–Denk bodies) found in patients with chronic liver disorders such as steatohepatitis and hepatocellular neoplasia.^27^ Therefore, it would be of great interest to investigate the properties of LCOs in labeling the multi-protein inclusion bodies observed in s-IBM.

Herein we report the usefulness of LCOs for the detection of multi-protein inclusion bodies in skeletal muscle fibers from s-IBM patients. Labeling with LCO resulted in intense fluorescence light emitted from the inclusion bodies, and LCOs with a specific molecular design also showed a variation in the spectral signature. LCOs with a backbone of at least five thiophene units detected very small inclusion bodies that were Congo Red and Thioflavin S (ThS) negative. Hence, LCOs are powerful tools that offer rapid and accurate detection of protein inclusion bodies and can be used to unravel the complexity of these disease-associated structures.

## Results and Discussion

### LCOs recognize inclusion bodies immunopositive for p62

**Figure 1:**
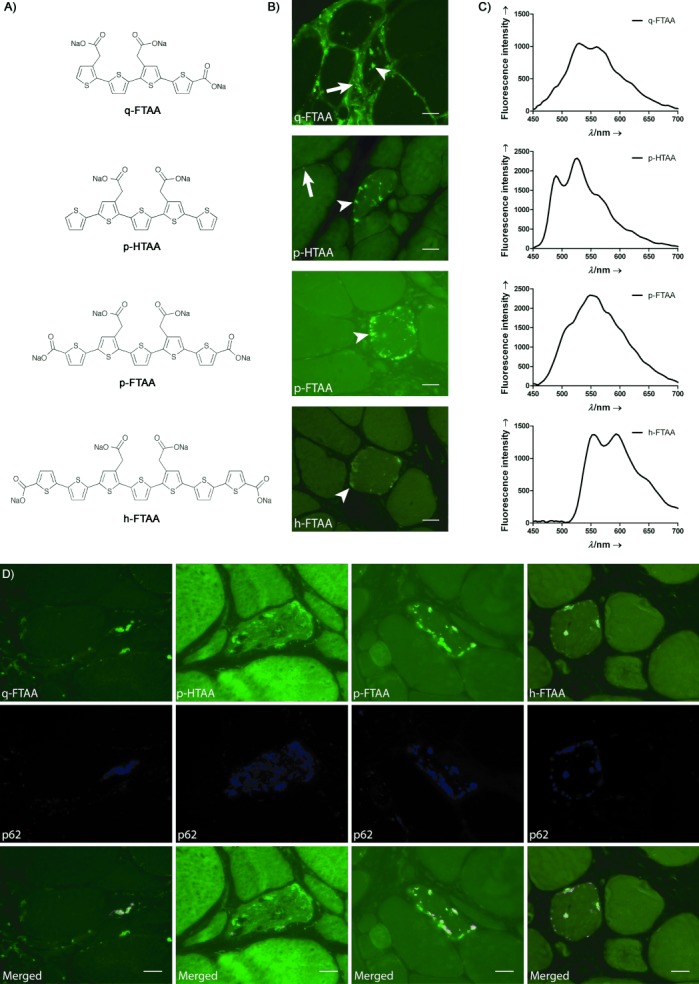
LCOs detect protein inclusion bodies in muscle tissue from s-IBM patients. A) Chemical structures of the LCOs q-FTAA, p-HTAA, p-FTAA, and h-FTAA. B) Fluorescence images of muscle tissue from s-IBM patient, stained with q-FTAA, p-HTAA, p-FTAA, or h-FTAA. LCOs bind to protein inclusion bodies (white arrowheads) in the muscle fibers and fluoresce with high intensity. White arrows identify autofluorescence from connective tissue or lipofuscin. C) Emission spectra of the indicated LCOs collected from s-IBM protein inclusion bodies defined in panel B. D) Fluorescence images of muscle tissue from s-IBM patients, stained with q-FTAA, p-HTAA, p-FTAA, or h-FTAA, together with an antibody against p62. All LCOs detected inclusion bodies immunopositive for p62. Scale bars: 20 μm.

**Figure 2:**
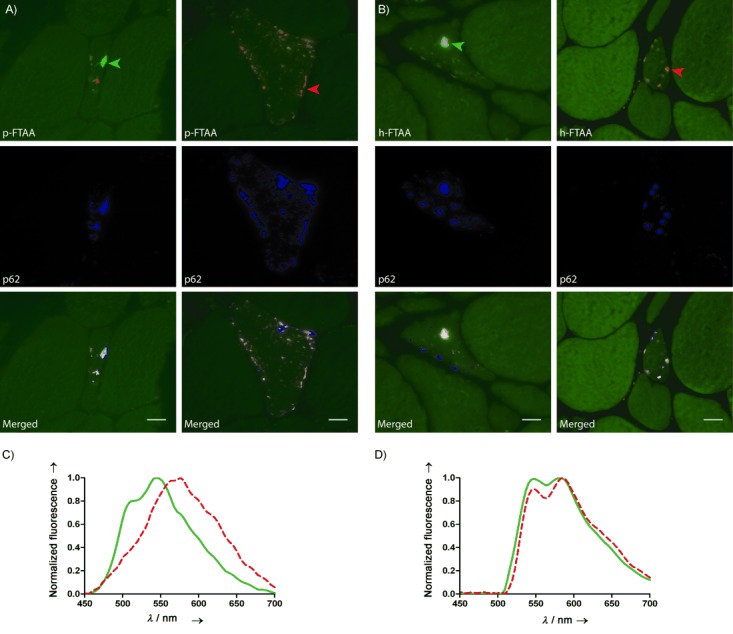
Fluorescence images and emission graphs of s-IBM muscle tissue stained with A) p-FTAA or B) h-FTAA, together with anti-p62 antibody. Binding to p62-positive inclusion bodies resulted in a variation in color emitted from p-FTAA or h-FTAA. C) p-FTAA emission spectra collected from p62-positive inclusion bodies in panel A: green arrowhead (green solid line); red arrowhead (red dashed line). The spectra reveal emission peaks at 544 and 572 nm respectively, thus confirming the difference in color observed for the inclusion bodies. D) h-FTAA emission spectra collected from p62-positive inclusion bodies in panel B; color coding as above. Both types of inclusion body display h-FTAA emission peaks at 548 and 585 nm; however, the lower intensity of the peak at 548 nm (inclusion marked with red arrowhead) confirms the color difference. Scale bars: 20 μm.

Earlier work has shown that LCOs bind to a plethora of protein aggregates including hepatic protein inclusion bodies termed Mallory–Denk.^9^, ^27^ To investigate whether LCOs could be utilized for general detection of protein inclusion bodies, we used muscle biopsies from s-IBM patients, as protein inclusion bodies in s-IBM muscle fibers have been reported to be composed of a variety of aggregated proteins.^18^–^24^ Biopsies from two to five s-IBM patients with p62-positive inclusion bodies were stained with the following LCOs: tetramer formyl thiophene acetic acid (q-FTAA), pentamer hydrogen thiophene acetic acid (p-HTAA), pentamer formyl thiophene acetic acid (p-FTAA), or heptamer formyl thiophene acetic acid (h-FTAA; Figure 1 A). All LCOs identified rounded or “squiggly” inclusion bodies (Figure 1 B) and displayed well-resolved emission spectra upon binding (Figure 1 C). The LCOs colocalized with an antibody against p62, which confirmed that the probes indeed labeled characteristic s-IBM protein aggregates (Figure 1 D, Figure 2). However, the ability to detect inclusion bodies was shown to be highly dependent on the molecular design of the LCO. The pentamers p-HTAA and p-FTAA and the heptamer h-FTAA showed strong positive staining in all p62-immunolabelled muscle fibers, whereas the tetramer q-FTAA only recognized a subset of these fibers. In addition, q-FTAA binding to inclusion bodies was only weakly fluorescent, and the emission signal was difficult to distinguish from autofluorescent structures (Figure 1 B), thus suggesting that a backbone of at least five thiophene units was necessary to identify all p62-positive muscle fibers. The molecular and photo-physical explanations for this observation are probably more complex. However, it was recently shown that tetrameric LCOs only detected mature amyloid fibrils during in vitro fibrillization of recombinant proteins, whereas LCOs with extended thiophene backbones (five to seven thiophene units) identified pre-fibrillar species (preceding the formation of amyloid fibrils).^12^ Thus, q-FTAA-negative inclusion bodies might represent protein inclusion bodies containing a less-amyloidogenic state of protein aggregates.

To verify our preliminary observations, we performed p-FTAA staining of additional s-IBM cases, and of non-s-IBM cases. Staining with p-FTAA showed the presence of p62 immunopositive inclusion bodies in 23 s-IBM samples (Table S1). The number of muscle fibers containing p-FTAA and p62-positive aggregates varied between patients, as did the morphology and size of the inclusion bodies. However, co-localization between p-FTAA and p62 antibody was consistently observed in individual muscle fibers for all cases. In 22 non s-IBM cases (controls; Table S1), double staining with p-FTAA and p62 antibody did not show any significant co-labeling (Figure S1). However, in four cases, some structures (Figure S2) were labeled with p-FTAA but not with p62 antibody. The number of muscle fibers containing these morphologies were always very scarce (one or two), and the structures were often found scattered in the sarcoplasm or just beneath the sarcolemma, in a focal plane other than that of the fiber. The same types of structures were also present in a small number of muscle fibers in four s-IBM cases. Muscle fibers containing protein inclusion bodies positive for both p-FTAA and p62 were always in large excess, thus suggesting that these structures were not associated with the diseases but had most likely arisen from the preparation of the tissue sections.

### Spectral assignment of s-IBM protein inclusion bodies

The initial experiments showed that pentameric and heptameric LCOs labeled p62-positive inclusion bodies in s-IBM muscle tissue, and that the inclusion bodies were easily identified as intense fluorescence from the probes. By using long-pass emission filters, it was also revealed that binding to aggregates in the muscle fibers resulted in a variation in color emitted from p-FTAA and h-FTAA. The observation was investigated in more detail by further assessing the color difference for p-FTAA by using a Spectraview device attached to the microscope, which allows collection of emission spectrum from a chosen area in the fluorescence image. In total, spectral variation was seen in 20 of the 23 p-FTAA-positive s-IBM cases that were analyzed by hyperspectral imaging. Excitation of p-FTAA at 436 nm and acquisition of a full-range emission spectrum (450–700 nm) confirmed a distinct variation in the spectral signatures upon binding to s-IBM protein inclusion bodies (Figure 2 A and C). The emission peaks for the majority of analyzed aggregates were found between 540 and 570 nm, but there were also inclusion bodies with green- or red-shifted spectra (emission maximum at 530 or 580 nm). Additional smaller peaks (or shoulders, depending on the spectral shift) were observed (510–520 and 610–620 nm). Spectral assessment of the heptameric LCO h-FTAA did not reveal the distinct separation between inclusion bodies shown with p-FTAA (Figure 2 B and D), and the phenomenon was completely lost when the carboxyl groups were removed from the 2- and 5′′′′-positions of the thiophene backbone (not shown). Hence, the number of thiophene units as well as the type of chemical end-group seem to be important structural aspects in the spectral separation of s-IBM inclusion bodies. The most distinct variation in color was achieved with a backbone of five thiopehene units and carboxyl moieties extending the conjugation length. The identical design has also been reported to spectrally distinguish between Aβ plaques and neurofibrillary tangles, the two major pathological hallmarks of AD.^8^, ^12^ Five thiophene units with carboxyl extensions of the backbone might constitute the optimal length for fitting in the binding pocket, while retaining the conformational freedom and conjugation length required for spectral discrimination of protein aggregates.

**Figure 3:**
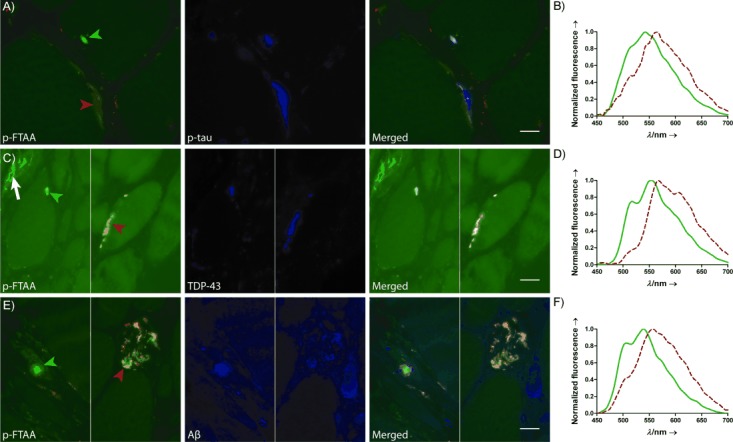
Fluorescence images and emission graphs for s-IBM muscle tissue stained with p-FTAA together with various antibodies. s-IBM protein inclusion bodies with antibodies against A) phosphorylated tau (p-tau), C) TDP-43, or E) amyloid β (Aβ) were labeled with p-FTAA, which resulted in both green (green arrowheads) and red (red arrowheads) emission from the LCO. The green structure in the left panel of C) is autofluorescence from connective tissue (white arrow). B) Emission spectra of p-FTAA (arrowheads in panel A). One emission maximum (green arrowhead) was at 542 nm (green solid line), whereas p-FTAA bound to elongated aggregate showed the highest intensity at 565 nm (red). D) Emission spectra from p-FTAA binding to inclusion bodies positive for TDP-43 (arrowheads in panel C). p-FTAA labeling the green-shifted inclusion (green arrowhead) showed maximum emission at 552 nm (green solid line). TDP-43 inclusion bodies with red emission from p-FTAA were only seen occasionally and are here represented by extremely red-shifted assemblies (greatest emission at 568 nm, additional peak around 610 nm; red dashed line). F) Emission spectra collected from p-FTAA bound to inclusion bodies positive for Aβ (arrowheads in panel E). The emission peak was at 540 nm for the green-shifted inclusion (green) and at 556 nm for the red-shifted aggregate (red). Scale bars: 20 μm.

As variation in the spectral fingerprint of p-FTAA bound to protein aggregates has previously been attributed to protein identity,^8^, ^12^ we next investigated whether the same explanation was applicable to s-IBM inclusion bodies. Muscle sections with s-IBM pathology were stained with antibodies against different proteins known to be part of s-IBM aggregates (Figure 3). As mentioned above, the majority of the inclusion bodies were immunoreactive for p62 antibody, and spectral analysis revealed that p-FTAA binding to p62-positive aggregates resulted in emission with both green- and red-shifted hue (Figure 2 A, 2C). Inclusion bodies with weak p62 staining were often green-shifted when labeled with p-FTAA. Additional analysis of sections co-stained with p-FTAA and antibodies against phosphorylated tau (Figure 3 A and B), TDP-43 (Figure 3 C and D), or Aβ (Figure 3 E and F) confirmed that the spectral variation could not be correlated with protein identity, as all the proteins were found in both green and red aggregates. The presence of TDP-43 often resulted in green p-FTAA emission, but red-shifted inclusions were also found occasionally. Hence, we concluded that the distinct spectral signature from p-FTAA for different inclusion bodies was most likely not due to the presence of a specific protein in the protein inclusion bodies.

**Figure 4:**
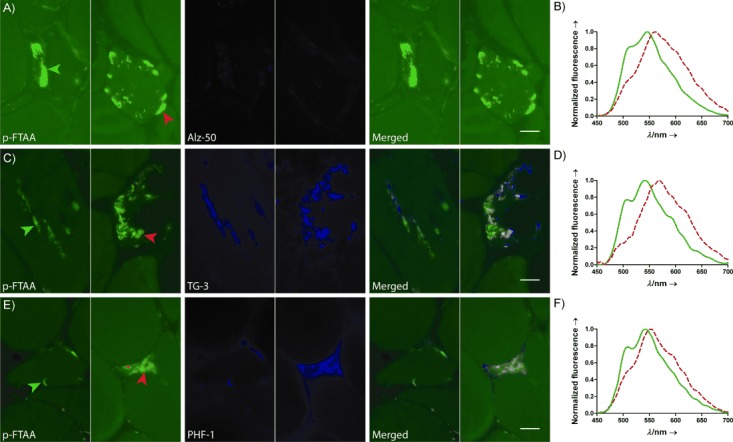
s-IBM muscle sections stained with p-FTAA and antibodies against conformation- and/or phosphorylation-dependent epitopes of tau. A) The conformational dependent antibody Alz-50, which binds an epitope exposed at the early stages of tau fibrillation, labeled neither green (green arrowhead) nor red (red arrowhead) p-FTAA-positive protein inclusion bodies. B) Emission spectra of p-FTAA binding to the protein inclusion bodies in panel A. The spectral graph confirms the observed variation in emission from p-FTAA and illustrates that Alz-50 epitope does not influence the LCO color. C) The conformation- and phosphorylation- dependent tau antibody TG-3 showed abundant staining of inclusion bodies in s-IBM muscle tissue. The antibody co-localized with p-FTAA, and the presence of the TG-3 epitope resulted in both green (green arrowhead) and red-shifted (red arrowhead) emission from the LCO. D) p-FTAA emission spectra collected from the TG-3-positive inclusion bodies in panel C. The emission peaks are at 542 nm and 570 nm, and the difference confirms the spectral variation of p-FTAA when bound to inclusion bodies exposing the TG-3 epitope. E) The phosphorylation-dependent epitope of PHF-1 was detected in a very small number of s-IBM protein inclusion bodies. p-FTAA labeling of PHF-1-positive tau aggregates resulted in both green (green arrowhead) and red (red arrowhead) color from the LCO. F) Emission spectra of p-FTAA collected from PHF-1 positive inclusion bodies in panel E. Presence of the PHF-1 epitope resulted in both green and red-shifted emission from p-FTAA (peaks at 540 and 552 nm, respectively). Scale bars: 20 μm.

As earlier work with prion deposits showed a correlation between variation in p-FTAA emission and protein conformation diversity,^8^ the spectral differences observed from distinct s-IBM inclusion bodies might reflect different aggregated states of a specific protein. Antibodies recognizing conformation-dependent epitopes of tau (Alz-50)^28^–^30^ only or in conjunction with site-specific phosphorylation (TG-3)^31^, ^32^ were applied to s-IBM muscle sections together with p-FTAA, to examine whether the spectral difference observed with the probe could be explained in terms of the structural heterogeneity of tau, as for the prion protein. The examined p-FTAA-positive protein inclusion bodies were negative for Alz-50 (Figure 4 A and B); whereas TG-3-positive aggregates were commonly found irrespective of the p-FTAA emission spectrum (Figure 4 C and D). The third type of tau antibody used on the s-IBM sections was the phosphorylation-dependent PHF-1. PHF-1 binds to tau phosphorrylated at Ser396/404;^33^ this is considered to be a late epitope in the aggregation pathway of tau compared with those recognized by Alz-50 and TG-3.^34^ However, when applied to s-IBM muscle sections, PHF-1 showed very poor reactivity, and only two muscle fibers with inclusion bodies strongly immunoreactive for PHF-1 were observed (Figure 4 E). p-FTAA labeling of these inclusion bodies resulted in emission of green and red light, thus indicating that the LCO color was not affected by the presence of the late (PHF-1-recognized) epitope (Figure 4 F). Reduction of incubation time with primary antibody or/and addition of p-FTAA (competing for the antibody binding site) are possible explanations for the diminution of Alz-50 and PHF-1 immunoreactivity in this work compared to that in previous studies.^35^ Either way, the result revealed that the spectral separation of s-IBM protein assemblies labeled with p-FTAA was not attributable to conformational diversity of tau, thus suggesting that the effect on the thiophene backbone conformation and conjugation length upon binding is not determined by tau only, but instead the collective assembly of misfolded proteins.

### Comparison of LCO staining with conventional amyloid ligands

**Figure 5:**
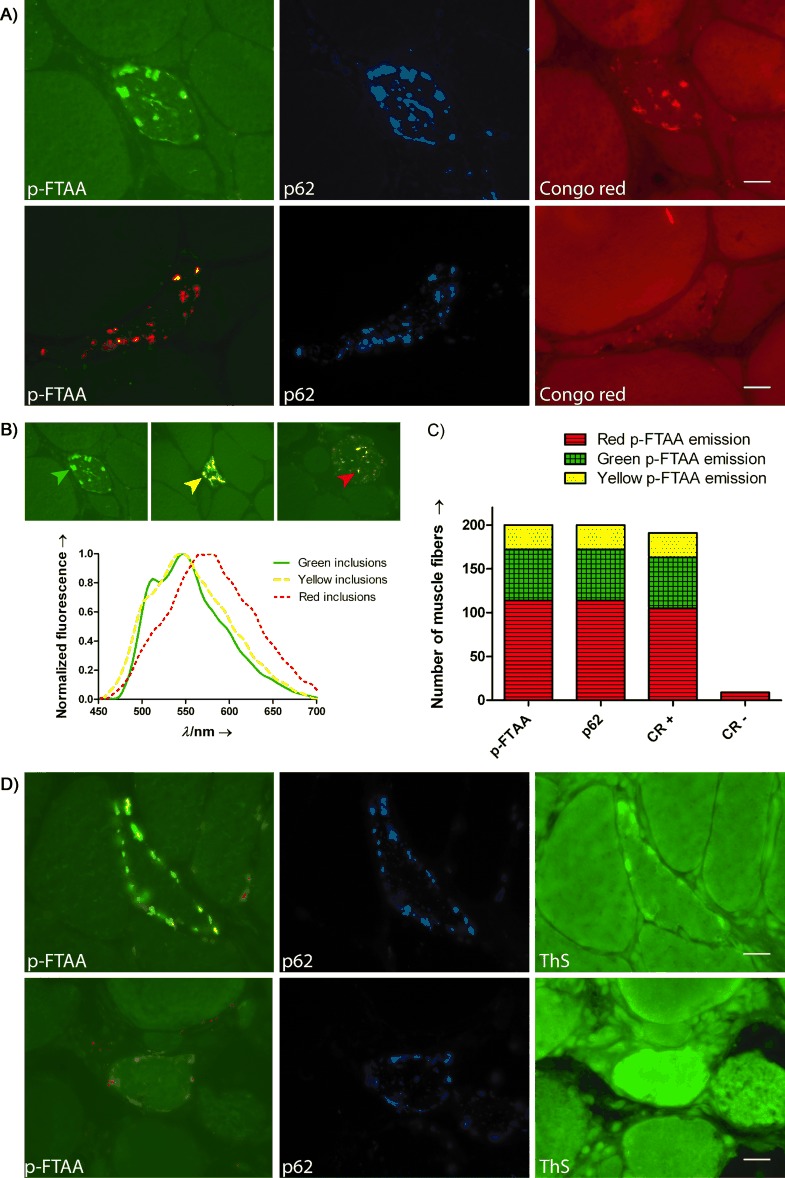
The detection of protein inclusion bodies in s-IBM muscle tissue by p-FTAA compared with the conventional amyloid-binding dyes Congo Red (CR) and ThS. A) Fluorescence images of consecutive muscle sections from s-IBM patient double-stained with p-FTAA and p62 antibody (left and middle) or single-stained with CR (right). When muscle fibers containing inclusion bodies (according to p-FTAA) were compared with the corresponding fibers in the CR section, it was evident that positive staining of CR could be seen irrespective of the color from p-FTAA. However, inclusion bodies with green-shifted emission from p-FTAA often showed higher fluorescence intensity from CR in the adjacent fiber compared to aggregates with red emission. B) Fluorescence images and p-FTAA emission spectra used to define inclusion bodies with green (green arrowhead, green solid line), yellow (yellow arrowhead, yellow dashed line) or red (red arrowhead, red dotted line) emission from p-FTAA. C) Analysis of 200 inclusion-containing muscle fibers for p-FTAA color (as defined in panel B), p62 immunoreactivity, and presence of CR fluorescence. Inclusion bodies with red-shifted emission from p-FTAA (red, striped area) were most abundant. p-FTAA and the antibody against p62 showed complete co-localization at the cellular level, whereas 4.5 % of the muscle fibers contained inclusion bodies positive for p-FTAA and p62 but negative for CR (CR-). All CR negative inclusion bodies displayed red emission from p-FTAA. D) Fluorescence images of consecutive s-IBM muscle section double-stained with p-FTAA and p62 antibody (left and middle) or single stained with ThS (right). ThS only detected a fraction of the inclusion bodies labeled with p-FTAA. Scale bars: 20 μm.

The staining pattern for the classic amyloid binding dye Congo Red on s-IBM muscle sections was compared with the result from p-FTAA together with an antibody against p62. The study included three consecutive sections from each of five s-IBM patients. The middle section was stained with Congo Red, and the surrounding two sections were stained with p-FTAA together with p62 antibody (Figure 5 A). In total, 200 muscle fibers were compared, and the results showed that the number of muscle fibers containing p-FTAA-positive inclusion bodies exceeded the number that were positive for Congo Red by 4.5 % (Figure 5 B and C). The difference was not likely caused by inclusion bodies disappearing because of their small size and the distance between two sections, as muscle fibers negative for Congo Red were positive for p-FTAA, both on the section preceding and the section following the one stained with Congo Red. Overall, detection of s-IBM protein inclusion bodies was easier with p-FTAA because of the higher quantum yield (the exposure time for p-FTAA was almost 10 times shorter than that for Congo Red), and p-FTAA allowed detection of very small aggregates.

In a similar fashion, we next compared the combined p-FTAA and p62 immunoprotocol with ThS staining by using adjacent sections from two s-IBM cases. In contrast to p-FTAA, ThS only showed partial co-localization with p62 staining at the cellular level (Figure 5 D). Analogous to the results obtained with the tetrameric LCO q-FTAA, ThS staining did not identify all p62-positive muscle fibers. Hence, p-FTAA identified a wider range of p62-positive protein inclusion bodies compared to the conventional amyloid ligands Congo Red and ThS. These findings are in agreement with previous studies that have shown that thiophene-based amyloid ligands detect a larger subset of extracellular protein aggregates in tissue sections than Congo Red and derivatives of thioflavin.^10^, ^36^–^39^ An earlier report^40^ also demonstrated a difference in the staining pattern obtained with ThS and an anti-Aβ antibody in s-IBM muscle tissue. ThS did not label all the immuno-histochemically detected Aβ, which either indicated the presence of non-aggregated Aβ or that ThS failed to detect some aggregated Aβ species. In the light of the LCO staining pattern, the latter seems more likely. The correlation between the immunological reactivity of the p62 antibody, a proposed clinical marker of s-IBM,^24^ and p-FTAA positivity in s-IBM muscle tissue, confirmed that the LCO-labeled structures were indeed protein aggregates. This could mean that p-FTAA yields stronger fluorescence signals than Congo Red or ThS, thus resulting in higher sensitivity and detection of deposits that would have gone undetected with the conventional probes. Alternatively, the range of aggregate topologies identified by p-FTAA might include a subset of those stained by Congo Red or ThS, as well as deposits not formally qualifying as amyloid. In vitro studies of recombinant proteins have also shown that LCOs with a repeating backbone of at least five thiophene units identify non-thioflavinophilic pre-fibrillar species that precede the formation of mature amyloid fibrils.^8^, ^12^, ^41^, ^42^ Whether the p-FTAA staining of inclusion bodies negative for Congo Red and ThS reflects its higher sensitivity or fundamental differences in the biophysical properties of the detected protein inclusion bodies is not known; however, the p-FTAA staining was restricted to s-IBM cases (Table S1) and to inclusion bodies that were stained by antibodies to proteins reported to be present in s-IBM inclusion bodies, thereby proving its high specificity.^18^–^24^

The Congo Red- and ThS-staining results also revealed that the color of p-FTAA did not determine whether the inclusion bodies were labeled with these classical amyloid ligands or not. These amyloid dyes could be seen binding to inclusion bodies in muscle fibers with green, yellow and red-shifted p-FTAA-positive aggregates on the adjacent section. However, there was a general tendency of inclusion bodies with a green color from p-FTAA to display stronger Congo Red and ThS fluorescence in the corresponding muscle fiber, compared to the red-emitting ones (Figure 5 A and D), and all muscle fibers negative for inclusion bodies according to Congo Red contained p-FTAA red-shifted aggregates (Figure 5 C). Hence, the red-shifted aggregates might represent less mature topologies of protein deposits not formally qualifying as amyloid. Overall, the p-FTAA spectral variation with s-IBM protein aggregates seems to mirror different types of inclusion bodies, and it was often possible to predict the color emitted from p-FTAA by looking at morphology and location. Histological samples from s-IBM patients only offer a snapshot of the stage of the disease, and development of s-IBM model systems is crucial to be able to further investigate the biophysical properties and possible maturation of these multi-protein inclusion bodies.

## Conclusions

In this study, LCOs were introduced as novel fluorescent tools for sensitive detection of multi-protein inclusion bodies in s-IBM muscle tissue. The pentameric LCO p-FTAA (carboxyl groups extending the conjugated backbone) was shown to be optimal, and the probe strongly highlighted protein inclusion bodies and enabled visualization of even very small aggregates that were negative for Congo Red and ThS. p-FTAA staining could also be used in combination with a diversity of antibodies. In addition, there was a distinct variation in the color emitted from p-FTAA upon binding to s-IBM inclusion bodies, and some results indicated that these spectral shifts might mirror distinct topologies of inclusion bodies. In conclusion, we have shown that p-FTAA is an excellent choice for rapid detection of protein inclusion bodies in muscle sections and that the probe can be used as a fluorescent tool to study these disease-associated structures.

## Experimental Section

**LCO synthesis and LCO staining**. The syntheses of q-FTAA, p-HTAA, p-FTAA and h-FTAA have been reported earlier.^8^, ^12^ Air-dried frozen muscle sections from s-IBM patients were preincubated for 10 min in PBS (phosphate buffer (10 mm, pH 7.4), NaCl (140 mm), and KCl (2.7 mm)), followed by 30 min incubation with q-FTAA, p-HTAA, p-FTAA, or h-FTAA (stock solutions: 1.5 mm in distilled water) diluted (1:500) in PBS. The sections were then washed with PBS and mounted with Vectashield Mounting Medium (Vector Laboratories, Burlingame, CA).

**Muscle biopsies**. Immuno-histochemical and spectral studies were performed on 10 or 12 μm sections of freshly frozen muscle biopsies, cryopreserved at −80 °C prior to experimental work-up and obtained with informed consent from 23 s-IBM patients at the Charité University Hospital, Berlin, Germany (*n*=15), the USC Neuromuscular Center at Good Samaritan Hospital, Los Angeles (*n*=5), and the University Hospital, Linköping (*n*=3). The median age of the s-IBM patients was 68 years (Table S1). The study also included muscle sections from patients diagnosed with polymyositis (*n*=2), dermatomyositis (*n*=3), amyotrophic lateral sclerosis (ALS, *n*=3), and polyneuropathy (*n*=3). As controls, we used muscle tissue from patients who, after all tests were performed, were considered free of muscle disease (*n*=11). The diagnosis of s-IBM was based on well-established criteria including Engel trichrome staining to identify vacuoles and inflammation, Congo Red staining with fluorescence enhancement to identify β-pleated sheet amyloid, and p62 to identify s-IBM inclusion bodies, complemented sometimes with antibody staining to identify other proteins commonly found in s-IBM inclusion bodies.

**Fluorescence microscopy and spectral analysis**. Fluorescence images and spectra were recorded with a DM6000 B fluorescence microscope (Leica Microsystems, Wetzlar, Germany) equipped with a SpectraCube module (Applied Spectral Imaging, Migdal Ha-Emek, Israel) with bandpass filters 436/10 (LP475), 480/20 (LP515), 535/50 (LP590), and 640/30 (BP 700/75). Spectra were collected from LCO-labeled inclusion bodies and are presented in the figures as the average of nine region of interest (ROIs) /muscle fiber with the background subtracted.

**Immunohistochemistry (antibody and LCO double-staining)**. As p62 has been reported as a specific marker of inclusion bodies in s-IBM,^24^ double staining with q-FTAA, p-HTAA, p-FTAA, or h-FTAA and an antibody against p62 was performed to confirm that the LCOs were labeling s-IBM inclusion bodies. Immunofluorescence with p62 antibody together with p-FTAA was performed on all muscle sections from patients diagnosed with s-IBM, polymyositis, dermatomyositis, ALS, or polyneuropathy, as well as on sections from controls (Table S1). The emission spectra of p-FTAA binding to protein inclusion bodies were analyzed in all 23 s-IBM cases. Air-dried sections were incubated for 30 min with either normal donkey or goat serum (5 %) to block nonspecific binding of secondary antibodies, followed by incubation with a mouse monoclonal anti-p62 antibody (1:100, BD Bioscience, Franklin Lakes, NJ) for 16 h at 4 °C in PBS. The sections were washed with PBS (3×5 min), and p62-labeled inclusion bodies were detected with donkey (1:200, Jackson ImmunoResearch Laboratories Inc, West Groove, PA) or goat (1:400, Thermo Fisher Scientific, Waltham, MA) anti-mouse secondary antibody conjugated with DyLight 649 or DyLight 650, respectively, for 1 h at RT. The sections were washed with PBS (3×5 min) and stained with LCOs as described above. The specificity of the secondary antibodies was examined by omitting the primary antibody in the staining procedure. Stained sections were analyzed with a Leica DM6000 B fluorescence microscope.

To investigate whether the spectral shift observed for protein inclusion bodies in s-IBM muscle tissue could be correlated with a specific protein accumulated in s-IBM muscle, sections were stained with p-FTAA in combination with one of the following: 1) mouse monoclonal antibody (AT100, 1:100; Thermo Fisher Scientific; *n*=18), which recognizes tau phosphorylated at Ser212 and Thr214 in Alzheimer’s brain tissue,^43^ 2) rabbit polyclonal anti-TDP-43 antibody (anti-TDP-43, 1:500; Proteintech Group Inc, Chicago, IL; *n*=18) or 3) mouse monoclonal anti-Aβ antibody (6E10, 1:100, Covance, Princeton, NJ; *n*=10). The staining protocol and microscope set-up were as for p62, with the exception of incubating the 6E10 antibody for 40 h at 4 °C and using a donkey anti-rabbit secondary antibody conjugated with DyLight 649 (1:200, Jackson ImmunoResearch Laboratories Inc) where appropriate.

Antibodies recognizing conformation-dependent epitopes of tau (previously shown positive in s-IBM muscle fibers)^35^ were used to examine whether the spectral shift seen in p-FTAA when binding to s-IBM inclusion bodies was dependent on conformationally modified tau. s-IBM muscle sections were stained with p-FTAA together with mouse monoclonal Alz-50 (*n*=2), TG-3 (*n*=5), or PHF-1 (*n*=6) antibody (all diluted 1:10, and generously provided by Dr. Peter Davies, Albert Einstein College of Medicine, New York, USA), by using the same immunohistochemical protocol and microscope set-up as described for p62.

**Comparison between Congo Red and p-FTAA staining**. Sections (10 μm) of freshly frozen muscle biopsies from five s-IBM patients were stained with Congo Red according to the method of Mendell et al.,^44^ and were viewed with a fluorescent microscope as described by Askanas et al.^25^ Sections adjacent to those stained with Congo Red were double-stained with p-FTAA and antibody against p62 as described above. The staining patterns for Congo Red, p-FTAA, and anti-p62 antibody were compared with a Leica DM6000 B fluorescence microscope with bandpass filter 560/40 (LP610) to evaluate Congo Red positivity, 480/20 (LP515) to investigate the color of the protein inclusion bodies and 640/30 (BP 700/75) to examine the presence of p62. Muscle fibers (total 200; 36–42 fibers from each patient) containing p-FTAA and p62 positive protein inclusion bodies were investigated.

**Comparison between ThS and p-FTAA staining**. Air-dried frozen muscle sections from two s-IBM patients were fixed in paraformaldehyde (4 %) for 10 min at RT, followed by methanol for 10 min at −20 °C. The sections were washed in distilled water and stained for 5 min at RT with ThS (; Sigma–Aldrich) dissolved (1 % *w*/*v*) in ethanol (50 %). Unbound ThS was removed with ethanol (50 %, 2×1 min) and distilled water (1×5 min). The sections were mounted with Vectashield Mounting Medium and analyzed with a Leica DM6000 B with bandpass filter 436/10 (LP475). The staining pattern of ThS was evaluated by using adjacent sections doubly labeled with p-FTAA and anti-p62 antibody, as described above.

## Supplementary material

As a service to our authors and readers, this journal provides supporting information supplied by the authors. Such materials are peer reviewed and may be re-organized for online delivery, but are not copy-edited or typeset. Technical support issues arising from supporting information (other than missing files) should be addressed to the authors.

## References

[1] Nilsson KPR (2009). FEBS Lett.

[2] Nilsson KPR, Herland A, Hammarström P, Inganäs O (2005). Biochemistry.

[3] Nilsson KPR, Hammarström P, Ahlgren F, Herland A, Schnell EE, Lindgren M, Westermark GT, Inganäs O (2006). ChemBioChem.

[4] Åslund A, Herland A, Hammarström P, Nilsson KPR, Jonsson B-H, Inganäs O, Konradsson P (2007). Bioconjugate Chem.

[5] Nilsson KPR, Åslund A, Berg I, Nyström S, Konradsson P, Herland A, Inganäs O, Stabo-Eeg F, Lindgren M, Westermark GT, Lannfelt L, Nilsson LNG, Hammarström P (2007). ACS Chem. Biol.

[6] Philipson O, Hammarstöm P, Nilsson KPR, Portelius E, Olofsson T, Ingelsson M, Hyman BT, Blennow K, Lannfelt L, Kalimo H, Nilsson LNG (2009). Neurobiol. Aging.

[7] Nilsson KPR, Ikenberg K, Åslund A, Fransson S, Konradsson P, Röcken C, Moch H, Aguzzi A (2010). Am. J. Pathol.

[8] Åslund A, Sigurdson CJ, Klingstedt T, Grathwohl S, Bolmont T, Dickstein DL, Glimsdal E, Prokop S, Lindgren M, Konradsson P, Holtzman DM, Hof PR, Heppner FL, Gandy S, Jucker M, Aguzzi A, Hammarström P, Nilsson KPR (2009). ACS Chem. Biol.

[9] Klingstedt T, Nilsson KPR (2011). Biochim Biophys Acta.

[10] Sigurdson CJ, Nilsson KPR, Hornemann S, Manco G, Polymenidou M, Schwartz P, Leclerc M, Hammarström P, Wütrich K, Aguzzi A (2007). Nat. Methods.

[11] Nilsson KPR, Joshi-Barr S, Winson O, Sigurdson CJ (2010). J. Neurosci.

[12] Klingstedt T, Åslund A, Simon RA, Johansson LBG, Mason JJ, Nyström S, Hammarström P, Nilsson KPR (2011). Org. Biomol. Chem.

[13] Goedert M, Spillantini MG (2006). Science.

[14] Vekrellis K, Xilouri M, Emmanouilidou E, Rideout HJ, Stefanis L (2011). Lancet Neurol.

[15] Lee VM, Goedert M, Trojanowski JQ (2001). Annu. Rev. Neurosci.

[16] Strnad P, Zatloukal K, Stumptner C, Kulaksiz H, Denk H (2008). Biochim. Biophys. Acta Mol. Basis Dis.

[17] Goldfarb LG, Dalakas MC (2009). J. Clin. Invest.

[18] Askanas V, Engel WK (2008). Acta Neuropathol.

[19] Askanas V, Engel WK, Alvarez RB (1992). Am. J. Pathol.

[20] Askanas V, Engel WK, Bilak M, Alvarez RB, Selkoe DJ (1994). Am. J. Pathol.

[21] Weihl CC, Temiz P, Miller SE, Watts G, Smith C, Forman M, Hanson PI, Kimonis V, Pestronk A (2008). J. Neurol. Neurosurg. Psychiatry.

[22] Askanas V, Engel WK, Alvarez RB, McFerrin J, Broccolini A (2000). J. Neuropathol. Exp. Neurol.

[23] Askanas V, Bilak M, Engel WK, Alvarez RB, Tomé F, Leclerc A (1993). Neuroreport.

[24] Nogalska A, Terracciano C, D’Agostino C, Engel WK, Askanas V (2009). Acta Neuropathol.

[25] Askanas V, Engel WK, Alvarez RB (1993). Neurology.

[26] Toyama BH, Weissman JS (2011). Annu. Rev. Biochem.

[27] Mahajan V, Klingstedt T, Simon R, Nilsson KPR, Thueringer A, Kashofer K, Haybaeck J, Denk H, Abuja PM, Zatloukal K (2011). Gastroenterology.

[28] Wolozin BL, Pruchnicki A, Dickson DW, Davies P (1986). Science.

[29] Hyman BT, Van Hoesen GW, Wolozin BL, Davies P, Kromer LJ, Damasio AR (1988). Ann. Neurol.

[30] Jicha GA, Bowser R, Kazam IG, Davies P (1997). J. Neurosci. Res.

[31] Vincent I, Rosando M, Davies P (1996). J. Cell Biol.

[32] Jicha GA, Lane E, Vincent I, Otvos L, Hoffman R, Davies P (1997). J. Neurochem.

[33] Otvos L, Feiner L, Lang E, Szendrei GI, Goedert M, Lee VM (1994). J. Neurosci. Res.

[34] Weaver CL, Espinoza M, Kress Y, Davies P (2000). Neurobiol. Aging.

[35] Nogalska A, D′Agostino C, Engel WK, Askanas V (2011). Neurosci. Lett.

[36] Maji SK, Perrin MH, Sawaya MR, Jessberger S, Vadodaria K, Rissman RA, Singru PS, Nilsson KPR, Simon R, Schubert D, Eisenberg D, Rivier J, Sawchenko P, Vale W, Riek R (2009). Science.

[37] Berg I, Nilsson KPR, Thor S, Hammarström P (2010). Nat. Protoc.

[38] Lord A, Philipson O, Klingstedt T, Westermark G, Hammarström P, Nilsson KPR, Nilsson LNG (2011). Am. J. Pathol.

[39] Schultz SW, Nilsson KPR, Westermark GT (2011). PLoS One.

[40] Schmidt J, Barthel K, Zschüntzsch J, Muth IE, Swindle EJ, Hombach A, Sehmisch S, Wrede A, Lühder F, Gold R, Dalakas MC (2012). Brain.

[41] Hammarström P, Simon R, Nyström S, Konradsson P, Åslund A, Nilsson KPR (2010). Biochemistry.

[42] Göransson AL, Nilsson KPR, Kågedal K, Brorsson AC (2012). Biochem. Biophys. Res. Commun.

[43] Zheng-Fishhöfer Q, Biernat J, Mandelkow E-M, Illenberger S, Godemann R, Mandelkow E (1998). Eur. J. Biochem.

[44] Mendell JR, Sahenk Z, Gales T, Paul J (1991). Arch. Neurol.

