# Supplementary material for: Luminescent Conjugated Oligothiophenes for Sensitive Fluorescent Assignment of Protein Inclusion Bodies
Source: Chembiochem. 2013 Feb 28;14(5):607–16. doi: 10.1002/cbic.201200731 (PMC3743175; doi:10.1002/cbic.201200731)
Supplement: Supplementary file 1 [file cbic0014-0607-SD1.pdf]

CHEMBIOCHEM

Supporting Information

© Copyright Wiley-VCH Verlag GmbH & Co. KGaA, 69451 Weinheim, 2013

Luminescent Conjugated Oligothiophenes for Sensitive Fluorescent Assignment of Protein Inclusion Bodies

Therése Klingstedt,[a] Cristiane Blechschmidt,[b] Anna Nogalska,[c] Stefan Prokop,[b] Bo Häggqvist,[d] Olof Danielsson,[d] W. King Engel,[c] Valerie Askanas,[c] Frank L. Heppner,[b] and K. Peter R. Nilsson*[a]

cbic_201200731_sm_miscellaneous_information.pdf

Supplemental Table 1: Clinical diagnosis and tinctorial properties of the included cases

Case number	*Diagnosis*	*Age/Sex*	*p62*	*p-FTAA*
1	s-IBM	78/M	+	+
2	s-IBM	62/M	+	+
3	s-IBM	56/M	+	+
4	s-IBM	65/M	+	+
5	s-IBM	65/M	+	+
6	s-IBM	55/M	+	+
7	s-IBM	70/F	+	+
8	s-IBM	74/M	+	+
9	s-IBM	74/F	+	+
10	s-IBM	53/M	+	+
11	s-IBM	41/M	+	+
12	s-IBM	81/F	+	+
13	s-IBM	70/M	+	+
14	s-IBM	74/F	+	+
15	s-IBM	49/M	+	+
16	s-IBM	68/M	+	+
17	s-IBM	72/M	+	+
18	s-IBM	64/M	+	+
19	s-IBM	68/M	+	+
20	s-IBM	60/F	+	+
21	s-IBM	58/M	+	+
22	s-IBM	73/M	+	+
23	s-IBM	82/F	+	+
28	Polymyositis	58/F	-	-
29	Polymyositis	50/F	-	-
30	Dermatomyositis	84/F	-	-
31	Dermatomyositis	57/F	-	-
32	Dermatomyositis	71/M	-	-
33	ALS	38/M	-	-
34	ALS	63/M	-	-
35	ALS	73/M	-	-
36	Polyneuropathy	61/F	-	-
37	Polyneuropathy	68/M	-	-
38	Polyneuropathy	50/M	-	-
39	Normal control	69/M	-	-
40	Normal control	80/M	-	-
41	Normal control	58/F	-	-
42	Normal control	43/M	-	-
43	Normal control	41/F	-	-
44	Normal control	30/F	-	-
45	Normal control	67/F	-	-
46	Normal control	80/M	-	-
47	Normal control	67/M	-	-
48	Normal control	66/F	-	-
49	Normal control	70/M	-	-

s-IBM; sporadic inclusion body myositis
ALS; amyotrophic lateral sclerosis
M; male
F; female

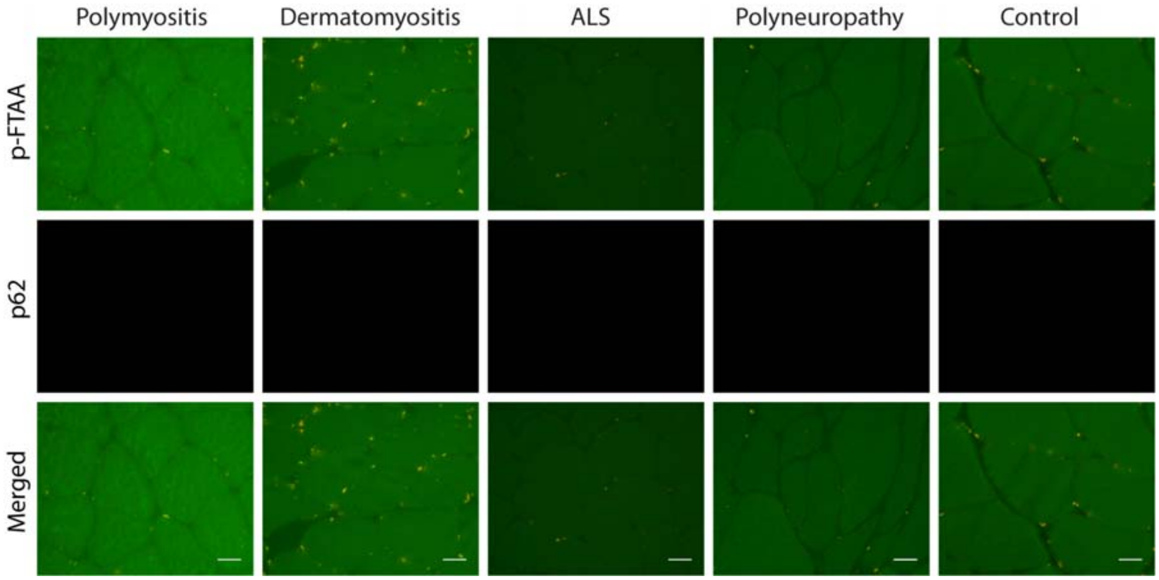

Supplemental Figure S1. Fluorescence images of muscle sections from patients with polymyositis, dermatomyositis, amyotrophic lateral sclerosis (ALS) or polyneuropathy as well as from control individual without muscle pathology double-stained with p-FTAA and antibody against p62. Scale bars represent 20 μm.

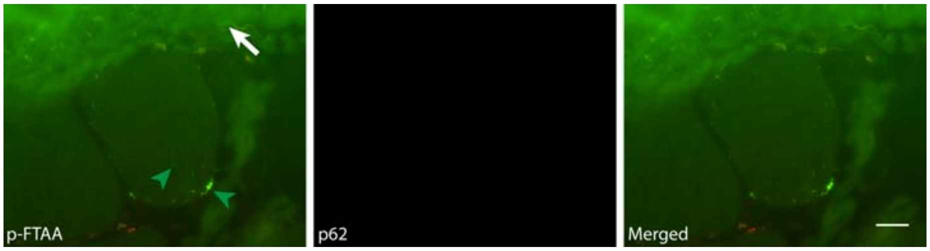

Supplemental Figure S2. Demonstration of structures in skeletal muscle fibers labeled with p-FTAA, but not p62 antibody. The structures were very small and often found in the subsarcolemmal region or scattered in the sarcoplasm (green arrowheads). The green structure in the top of the figure is autofluorescence from connective tissue (white arrow). Scale bar represents 20 μm.
